# *Staphylococcus aureus* Toxins: An Update on Their Pathogenic Properties and Potential Treatments

**DOI:** 10.3390/toxins13100677

**Published:** 2021-09-23

**Authors:** Nour Ahmad-Mansour, Paul Loubet, Cassandra Pouget, Catherine Dunyach-Remy, Albert Sotto, Jean-Philippe Lavigne, Virginie Molle

**Affiliations:** 1 Laboratory of Pathogen Host Interactions, CNRS UMR5235, Université de Montpellier, 34000 Montpellier, France; nour.mansour@umontpellier.fr; 2 Virulence Bactérienne et Infections Chroniques, INSERM U1047, Department of Infectious and Tropical Diseases, Université de Montpellier, 30908 Nîmes, France; paul.loubet@chu-nimes.fr (P.L.); albert.sotto@chu-nimes.fr (A.S.); 3 Virulence Bactérienne et Infections Chroniques, INSERM U1047, Université de Montpellier, 30908 Nîmes, France; cassandra.pouget@gmail.com; 4 Virulence Bactérienne et Infections Chroniques, INSERM U1047, Department of Microbiology and Hospital Hygiene, Université de Montpellier, 30908 Nîmes, France; catherine.remy@chu-nimes.fr (C.D.-R.); jean.philippe.lavigne@chu-nimes.fr (J.-P.L.)

**Keywords:** *Staphylococcus aureus*, pathogenicity, toxins, anti-toxin strategies, virulence

## Abstract

*Staphylococcus aureus* is a clinically important pathogen that causes a wide range of human infections, from minor skin infections to severe tissue infection and sepsis. *S. aureus* has a high level of antibiotic resistance and is a common cause of infections in hospitals and the community. The rising prevalence of community-acquired methicillin-resistant *S. aureus* (CA-MRSA), combined with the important severity of *S. aureus* infections in general, has resulted in the frequent use of anti-staphylococcal antibiotics, leading to increasing resistance rates. Antibiotic-resistant *S. aureus* continues to be a major health concern, necessitating the development of novel therapeutic strategies. *S. aureus* uses a wide range of virulence factors, such as toxins, to develop an infection in the host. Recently, anti-virulence treatments that directly or indirectly neutralize *S. aureus* toxins have showed promise. In this review, we provide an update on toxin pathogenic characteristics, as well as anti-toxin therapeutical strategies.

## 1. Introduction

*Staphylococcus aureus* continues to be one of the most involved bacteria in human diseases. This bacteria is found in the normal skin microbiota of both animals and humans, with a carriage rate between 20 and 30% in the healthy human population [1,2]. Abscesses, lung infections, bacteremia, endocarditis, and osteomyelitis are all caused by *S. aureus* infections in humans [3]. With the appearance of methicillin-resistant *S. aureus* (MRSA) strains, the pathogenicity of *S. aureus* has become a problem in both health institutions and community settings. MRSA is on the rise since its discovery in the early 1960s, although there has been some stabilization or decline in European countries [4]. However, MRSA remains an important opportunistic pathogen in Europe and the most frequently identified worldwide [5]. MRSA is prevalent in several hospitals, especially those in Europe, Asia, and the United States. The prevalence of CA-MRSA strains from community-acquired (CA) infections among previously healthy individuals with few or no traditional healthcare-associated (HA) risk factors for MRSA increased in the late 1990s.

*S. aureus* infections rely on the production of surface proteins that initiate bacterial adherence to host tissues, the secretion of extracellular toxins and enzymes that destroy host cells and tissues, the avoidance or inactivation of the host immune system, and the growth and expansion of bacteria in host cells and tissue [6]. Coagulase, hyaluronidase, deoxyribonuclease, and lipase are some of the enzymes that *S. aureus* can synthesize to enhance its pathogenicity and disseminate within the host [7]. Moreover, enterotoxins, toxic shock syndrome toxin 1 (TSST-1), exfoliative toxins (ETs), hemolysins, epidermal cell differentiation inhibitors (EDINs), and Panton–Valentine leukocidin (PVL) have all been identified as extracellular protein toxins that enhance pathogenicity [8]. Interestingly, some of these toxins were detected in MRSA infections more frequently than non-MRSA cases [9,10,11].

Hospitalizations related to staphylococcal infections are frequent, increasing mortality and health costs [12,13]. Moreover, *S. aureus’* capacity to produce antibiotic-neutralizing enzymes has exacerbated the issues associated with antimicrobial therapy, resulting in numerous resistances to these drugs [14]. Antibiotic resistance enzymes play a significant role in bacterial resistance to antibiotic pressure regarding diversity, evolution, and spread. Antibiotic-producing bacteria need strategies to counteract the chemicals’ deadly effects, by the production of degradative enzymes [14,15]. However, the selection pressure caused by the widespread use of antibiotics in humans and animals propagated resistant bacterial clones.

Antibiotic resistance develops quickly in *S. aureus*, and the rise of multidrug-resistant forms is a major problem. It has been reported that the annual mortality toll from antibiotic-resistant diseases has surpassed 10 million and that by 2050, it will outnumber cancer deaths [16]. The morbidity and mortality consequences reinforce the need to urgently discover new effective solutions due to the inefficiency of traditional antibiotics. Therefore, alternative treatments represent a promising field of investigation due to the lack of new antibiotic classes. Different strategies have been conducted, notably based on drug design with synthetic analogs, that could inhibit virulence factors. However, these studies have not yet generated promising results due to toxicity and/or low bioavailability. New options are now under study with a focus on biological molecules or compounds to interfere with toxins or toxin-regulator genes, constituting a new generation of promising anti-staphylococcal treatments [17,18,19,20,21].

This review outlines key properties related to the pathogenic roles of numerous *S. aureus* toxins (Table 1), as well as up to date anti-toxin treatments (Table 2).

## 2. Toxins Involved in the Pathogenicity of *S. aureus*

### 2.1. Staphylococcal Pore-Forming Toxins (PFTs)

PFTs are a type of bacterial virulence factor found in a wide range of human diseases, including *S. aureus*, which uses a variety of pore-forming cytotoxins (i.e., hemolysins, leukotoxins, and phenol-soluble modulins) to create pores in the host cell membrane causing cell lysis or to disrupt host cell actin cytoskeleton creating breaches in endothelial cells (EDIN exotoxin).

#### 2.1.1. Hemolysins

*S. aureus* encodes α-, β-, γ-, and δ-hemolysins, which are regulated by the accessory gene regulator (Agr) and, principally, lyse erythrocytes by creating pores in host cell membranes or dissolving cell wall components [22]. The best-studied virulence factor of *S. aureus* is α-hemolysin, encoded by the *hla* gene, causing damage to a large variety of host cells, such as epithelial cells, endothelial cells, erythrocytes, monocytes, and keratinocytes, as well as causing cell membrane damage and apoptosis [22]. It is the prototype for the small β-barrel class pore-forming cytotoxins that is secreted as a 33 kDa water-soluble monomer that forms a prepore by assembling into one homoheptamer. Then, this prepore matures as a β-barrel transmembrane aqueous channel [23]. Finally, the binding of α-hemolysin to its host receptor ADAM10 stimulates ADAM10’s metalloprotease activity, allowing it to cleave endothelial cadherin, compromising endothelial barrier function [24]. Cellular reactions, including the release of potent lipid mediators originating from the arachidonate cascade, are then activated by the transport of ions such as Ca2+ through the pore, resulting in the target cells apoptosis [25].

The large majority of *S. aureus* strains (95%) possess the *hla* gene, irrespective of their resistance to methicillin, without showing a specific repartition in *S. aureus* clones nor a higher prevalence in certain regions of the world [26]. The role of the *α*-hemolysin toxin in the development of severe infections, such as pneumonia, osteomyelitis, and bacteremia, has been established in studies employing different experimental models infected with the *S. aureus* USA300 strain [27,28]. Interestingly, even though most recent *S. aureus* strains encode *hla*, data suggest that greater *hla* expression promotes pathogenicity. For instance, in a rat model of pneumonia, the epidemic strain USA300, which supplanted USA400 to become the dominant community-acquired methicillin-resistant *S. aureus* (CA MRSA) strain in the United States during the early 2000s, was reported to be significantly more virulent and fatal than USA400 and was strongly correlated with a substantial increase in *hla* expression [27,29].

Therefore, based on its crucial role in virulence, the *α*-hemolysin toxin is an ideal target for the development of anti-toxin treatments against *S. aureus*.

#### 2.1.2. Panton-Valentine Leukocidin (PVL)

Leukotoxins target white blood cells, such as neutrophils, monocytes, or macrophages [30]. Panton–Valentine leukocidin (PVL), LukDE, and LukAB (sometimes known as LukGH) are all members of the bi-component Luk toxin family, with PVL presenting a 100-fold higher leukocytotic activity than the others. This Luk toxin family includes 32–35 kDa leukotoxins, which are encoded on the core genome or phage and oligomerize to form a pore structure [31]. Leukotoxins’ leukocytotic activity is based on receptor interaction. CCR5 on immune cells is the receptor for LukDE, whereas C5aR, C5L2, and CD11b are the receptors for PVL and LukAB [32,33,34]. PVL is a toxin that is made of two parts: LukS-PV and LukF-PV. These two components are excreted before assembling into a pore-forming heptamer on neutrophil membranes, resulting in their lysis [35].

PVL is primarily linked to skin and soft tissue disease, with other types of invasive disease, such as pneumonia, musculoskeletal disease, and bacteremia, being far less common. Infection with a PVL-positive strain does not appear to predict a poor clinical outcome for staphylococcal pneumonia, musculoskeletal disease, or bacteremia in adults, but patients with PVL-positive skin and soft tissue disease seem to be more likely to require surgical intervention [36]. PVL is linked to skin and soft tissue infections in both MRSA and MSSA strains, irrespective of the strain type [36,37,38].

Therefore, new treatments are required because the likelihood of infection with PVL-positive *S. aureus* strains is rising, and some of these strains are MRSA that already have limited treatment options.

#### 2.1.3. Phenol-Soluble Modulins (PSMs)

PSMs are one of the most important and aggressive virulence factors in *S. aureus* involved in a variety of staphylococcal pathogenesis, such as red and white blood cell lysis, inflammatory response induction, and antimicrobial activities [39,40,41]. Moreover, while PSMs are reported to be the most cytolytic and immunological modulating factors, they all play a function in epithelial surface spreading and have also been associated with the structuring and detachment of biofilms [42,43]. *S. aureus* produces a variety of PSMs, each with unique cytolytic and antibacterial characteristics [41]. These toxins are a class of small peptides with an amphipathic α-helical structure and surfactant-like characteristics [41]. PSMs are categorized into two subfamilies: (i) PSMα peptides that are 20–26 amino acids long and contain PSMα1–PSMα4 and the δ-toxin and (ii) PSMβ peptides that are 43–44 amino acids in length and contain PSMβ1 and PSMβ2 [39]. The PSMα and β peptides are encoded in the *psmα* *and psm*β operon, while the δ-toxin gene is within the sequence of RNAIII, the effector molecule of the Agr (accessory gene regulator) quorum-sensing pathway [44,45]. PSMs attach to the formyl peptide receptor 2 (FPR2), which attracts innate immune cells, such as neutrophils, macrophages, and dendritic cells [46,47]. As a result, holes formed in the host cell membrane cause osmotic instability and cell lysis. PSMα peptides have shown their great capacity to lyse human leukocytes and erythrocytes, with PSMα3 having the most important activity. The δ-toxin, on the other hand, has a mild cytolytic activity, while the PSMβ peptides are non-cytolytic [40].

PSMs are mainly present in highly virulent *S. aureus*, notably CA-MRSA. In vitro, these strains show a greater expression of PSMs, particularly cytolytic PSMα peptides, than that of hospital-acquired MRSA (HA-MRSA) strains [48]. In animal infection models, the PSMα peptides generated by the CA-MRSA USA300 and USA400 have a significant impact on the ability of virulent *S. aureus* to generate cutaneous infection and bacteremia [49,50,51].

Therefore, targeting PSMs for anti-staphylococcal treatment and drug development would be beneficial since eliminating all PSMs’ cytolytic and pro-inflammatory activities would lower their potency against host cells and possibly their overall contribution to *S. aureus* disease progression.

#### 2.1.4. Epidermal Cell Differentiation Inhibitor (EDIN) Exotoxins

To date, three forms of EDIN toxins have been identified: EDIN-A, EDIN-B, and EDIN-C [52,53]. EDINs enter host cells and induce macroapertures, which are large and temporary transcellular tunnels within endothelial cells, thus compromising the integrity of the endothelium barrier, and then target and inhibit the small host protein RhoA [54,55]. This small GTPase is a critical regulator of the actin cytoskeleton in the host cell [56]. The inhibition of RhoA has been shown in several cell biology studies to have a negative effect on the cohesiveness of the epithelial and endothelium barrier, thus favoring bacterial spread [54,57]. In addition, RhoA inhibition suppresses complement-mediated phagocytosis [58]. Overall, a significant number of research exploring the effects of RhoA inhibition indicate that EDINs secreted factors play an important role in *S. aureus* colonization and bacterial host tissue invasion [59,60].

Numerous pathogenic strains of *S. aureus*, especially those from the European MRSA lineage (ST80-MRSA-IV) [53], express EDIN or EDIN-like exotoxins [61,62,63]. The prevalence of these genes in *S. aureus* is poorly described. Though, in diabetic foot ulcers, *S. aureus* isolates were positive for *edin* (A and B) genes in 14 (7.2%) out of 195 patients [53]. A prevalence of 14 % of EDIN-encoding genes (primarily *edinC*) was found in 256 *S. aureus* isolates from diverse clinical sites of infection in Nice (France) [64]. Interestingly, the association between PVL and EDIN among MRSA has been observed with a prevalence varying between 12 and 100% [65].

EDIN exotoxins are thus important virulence factors in promoting bacterial colonization and host tissue invasion, such as in diabetic foot infections, bacteremia, and pneumonia [53,66,67].

### 2.2. Exfoliative Toxins (ETs)

Staphylococcal exfoliative toxins (ETs) are responsible for staphylococcal scalded skin syndrome (SSSS), also known as Ritter’s disease, and characterized by dehydration, the loss of superficial skin layers, and secondary infections [68]. Large areas of the body are affected by SSSS, and the lesions are frequently sterile. Bullous impetigo is a skin disease caused by the same exfoliative toxin that causes SSSS, generated by the same underlying infection, and most commonly affects the face, hands, trunk, and buttocks. Pustules and blisters grow near the original site of infection in bullous impetigo but not elsewhere on the body as in SSSS. Because the blisters and pustules originate so close to the epidermis’ surface, they never grow larger than a few millimeters before perforating and expanding at the border, where oozing and yellow crusting develop, and the infection might disseminate to the surrounding skin when patients rub the rash [69]. Therefore, the only difference between the two disorders is the level of skin damage. Bullous impetigo usually affects young children and infants, although redness and rashes do not develop as they would in SSSS because older children and adults possess neutralizing antibodies that inactivate the toxin [69]. SSSS also primarily affects newborns and infants, although it can also impact adults with renal insufficiency or immunological deficiencies [68]

ETA, ETB, ETC, and ETD are the most common ETs, while ETA and ETB have received most of the attention due to their link to SSSS [68]. ETs are encoded on various genetic elements, and their expression is controlled by the accessory gene regulator (Agr) [31,70]. ETs exhibit glutamate-specific serine protease activity and target desmoglein 1 (Dsg1; desmosomal intercellular adhesion molecule), a keratinocyte cell–cell adhesion protein [71]. ETs bind to Dsg1, destroying desmosomal cell attachments and causing epidermal dissociation of the human epidermis [72]. The rupture of epidermal layers allows bacteria to penetrate the skin and induce blistering disorders, such as bullous impetigo and SSSS [73].

The prevalence of ETA in methicillin-resistant (MRSA) and methicillin-susceptible (MSSA) strains does not differ considerably. According to recent studies, 4% of MSSA strains possess the *eta* or *etb* gene, while about 10% of MRSA strains are *eta* positive [74,75]. Resistant strains, however, may pose a problem in the future. For instance, in Japan, issues with treating *etb*-positive CA-MRSA infections that causes SSSS in healthy persons have already been described [76,77].

### 2.3. Superantigens (SAgs)

*S. aureus* superantigens (SAgs) are the most effective T-cell mitogens. The mechanism of action of SAgs varies from those of traditional peptide antigens. Antigen-presenting cells (APCs) ingest and process conventional antigens [78]. T-cells are able to identify an MHC class II-restricted antigenic peptide exposed on APC surfaces utilizing hypervariable regions of T-cell receptor (TCR) *α*- and *β*-chains [78]. However, SAgs can directly link TCR *β*-domains by exploiting conserved MHC class II structures displayed on APCs, then triggering T-cell activation and proliferation without the use of antigen processing [79]. This causes pro-inflammatory cytokines, including IL-2, IFN-γ, and TNF-α, to become overactive and release causing a multitude of side effects and symptoms, including the possibility of multi-system organ failure that is specific to each superantigen [79].

SAgs include staphylococcal enterotoxins (SEs) that have emetic effects after oral administration and the toxic shock syndrome toxin 1 (TSST-1) that does not have emetic properties [80].

#### 2.3.1. Staphylococcal Enterotoxins (SEs)

SEs are 20–30 kDa released toxins that disrupt intestinal activity and induce staphylococcal food poisoning (SFP), which is characterized by nausea, vomiting, abdominal pain, and diarrhea without indications of toxic effects, such as fever or hypotension [81,82,83]. Based on antigenic heterogeneity, more than 20 SEs (SEA—SElV) have been discovered [81,84,85]. Although the receptors involved in the emetic response to SEs have not been discovered, clinical signs of SFP have been linked to inflammatory mediators, such as leukotriene B4 and prostaglandin E2, both of which are produced in response to SEs [86,87]. The stomach and upper small intestine present the most significant mucosa lesions, which are associated with neutrophil infiltrates in the epithelium and lamina propria, whereas the jejunum exhibits broken brush boundaries and enlarged crypts [88].

In some CA-MRSA infections, lethal sepsis, infective endocarditis, and kidney infections are critically dependent on a high level of staphylococcal enterotoxin C (SEC) [89]. While staphylococcal enterotoxin B (SEB) is associated with food poisoning, it has been studied for potential utilization as an inhaled biological weapon [88].

#### 2.3.2. Toxic Shock Syndrome Toxin 1 (TSST-1)

Unlike SEs, TSST-1 (22-kD) does not trigger emesis but stimulates the release of substantial amounts of pro-inflammatory cytokines from the host T-cells and macrophages [90]. This cytokine outburst causes toxic shock syndrome (TSS) symptoms, such as high fever, rash, desquamation, hypotension, and hypovolemic shock, which can progress to multiorgan failure [91].

Given the growing development of MRSA infections associated with TSST-1 expression, it is therefore becoming more difficult to treat and may ultimately lead to death.

**Table 1:** Toxins secreted by *S. aureus*.

Toxin	Biological Properties and Function	Associated Disease	References
α-hemolysin	- Pore-forming activity - Lysis of erythrocytes, leukocytes, epithelial cells, and fibroblasts - Pro-inflammatory properties	- Pneumonia - Sepsis	[23,24,92,93]
Panton–Valentine Leukocidin (PVL)	- Pore-forming activity - Lysis of neutrophils, monocytes, macrophages - Pro-inflammatory properties	- Pneumonia - Bacteremia - Necrotizing fasciitis - Skin and soft tissue infections	[30,31,94]
Phenol-Soluble Modulins (PSMs)	- Pore-forming activity - Lysis of erythrocytes, neutrophils, monocytes, bacterial protoplasts, spheroplasts - Pro-inflammatory properties - Promote biofilm formation	- Bacteremia - Skin infection	[40,82,95,96]
Epidermal Cell Differentiation Inhibitor (EDIN)	- Transcellular tunnel activity - Breaches in endothelial cells	- Pneumonia - Bacteremia - Diabetic foot ulcer	[53,54,66]
Exfoliative Toxins (ETs)	- Serine protease activity - Disruption of the cell–cell adhesions and junctions of the epidermis cells	- Staphylococcal scalded skin syndrome (SSSS)	[68,97]
Staphylococcal Enterotoxins (SEs)	- Superantigen activity - Pro-inflammatory activity	- Staphylococcal food poisoning - Toxic shock syndrome	[19,81,85]
Toxic Shock Syndrome Toxin 1 (TSST-1)	- Superantigen activity - Pro-inflammatory activity	- Toxic shock syndrome	[82,90,91]

## 3. Anti-Toxin Treatments

The growth and spread of antibiotic resistance among *S. aureus* strains emphasize the imperative need for the development of alternative treatments that do not exert selective pressure in order to avoid evolution toward multi-resistance, such as that experienced with antibiotics. Interestingly, toxin-targeting therapy has already been effective against a variety of pathogenic bacteria, including *S. aureus* [17]. The therapeutic treatments that neutralize or interfere with the expression of staphylococcal toxins are detailed in this section.

### 3.1. Antibodies

Antibodies are one of the most important anti-virulence strategies for neutralizing toxins. Unlike active immunization, which would necessitate multiple boosters and a lengthy time to produce optimal immune responses, passive immunization would give prompt treatment for infected patients, thereby reducing the severity of *S. aureus* infections.

For instance, attempts to neutralize the α-hemolysin toxin during the course of human infection are underway and are based on substantial evidence for its participation in pathogenesis in murine models, as well as its putative importance in human disease. MEDI4893 (suvratoxumab), an α-hemolysin-neutralizing monoclonal antibody (mAb) formerly called LC10, is among the best-studied anti-virulence treatments against *S. aureus* infections [21]. As described above, α-hemolysin interacts with the metalloprotease ADAM10, which promotes oligomerization and pore formation [98]. By binding to a highly conserved area of the α-hemolysin toxin, MEDI4893 inhibits its interaction with ADAM10 as well as it self-oligomerization, thus neutralizing its action [98,99,100]. The treatment of rabbits with MEDI4893 resulted in a considerable reduction in clinical outcomes, according to Le et al. [101]. Similarly, Ortines et al. found that *S. aureus*-infected mice previously passively immunized with MEDI4893 showed fewer wounds and decreased bacterial counts than those of untreated controls in non-diabetic and diabetic mice [102]. Surewaard et al. recently revealed that α-hemolysin causes rapid platelet aggregation and liver injury, resulting in multi-organ failure during *S. aureus* sepsis, but these consequences could be avoided in mice treated with MEDI4893 [103]. MEDI4893 has passed Phase 2 clinical studies for the prevention of *S. aureus* pneumonia in high-risk critical care unit patients in 2020, and more recently, MEDI4893 showed efficacy and safety in preventing *S. aureus* ventilator-associated pneumonia [104,105]. In addition, AR-301 and ASN100 are two other neutralizing antibodies that have entered clinical trials. AR-301 is an α-hemolysin-targeting monoclonal antibody that has recently entered Phase 3 tests as an adjuvant therapy for *S. aureus* pneumonia [106], and ASN100 is a combination of two monoclonal antibodies that neutralize six cytolytic toxins corresponding to α-hemolysin, PVL, LukAB, γ-hemolysin AB (HlgAB), γ -hemolysin CB (HlgCB), and leukocidin ED (LukED) [107]. Unfortunately, while ASN100 passed the Phase 1 clinical safety testing by reducing tissue damage in a rabbit model of *S. aureus* pneumonia, the Phase 2 trial was stopped due to inefficiency [107]. Although these trials involving MEDI4893 [108], AR-301 [106], and the multivalent antitoxin ASN100 [107] were not statistically significant, passive immunization was found to have some protective potential. For example, AR-30 shortened the time spent on mechanical ventilation, whilst MEDI4893 decreased hospital and intensive care unit stay, as well as antibiotic treatment duration, while remaining safe and well tolerated [106,107,108]. Foletti et al. identified antibodies against α-hemolysin from a human donor-derived single-chain variable fragment (scFv) phage library [99]. LTM14, a notable clone in this family, was transformed to a complete IgG and showed an unusually high affinity for α-hemolysin. LMT14 offered protection against *S. aureus* cutaneous and bacteremia mice models of infection and also demonstrated therapeutic potential in a pneumonia model [99]. In addition, when combined with the antibiotic linezolid, LTM14 showed improved efficacy. This is essential because, in a therapeutic situation, passive immune treatment will almost certainly be delivered in conjunction with the most suitable antibiotic [99].

While some studies have found an epidemiological link between PVL and CA-MRSA, the presence of large levels of neutralizing antibodies did not provide resistance to PVL-positive MRSA skin and soft tissue infections [109,110,111]. Despite this, PVL is still one of the most important targets for anti-toxin drug research. PVL-specific antibodies are present in available commercial human intravenous polyclonal immunoglobulin preparations (IVIg), which decrease the cytopathic effects of PVL in a dose-dependent manner, most likely through interfering with PVL–neutrophil interactions [112]. In 2015, Mairpady Shambat et al. showed that IVIg abolished PVL and α-hemolysin-mediated cytotoxicity in epithelial cells in a human lung tissue model [113]. Moreover, antibiotic therapy combined with IVIg anti-toxin treatment significantly improved the situation of patients with acute necrotizing pneumonia caused by PVL-positive *S. aureus* strains, demonstrating the effectiveness of IVIg in limiting disease progression, particularly in highly lethal *S. aureus* infections linked to PVL production [114]. Moreover, in vitro humanized antibodies developed by Leventie et al. are able to disrupt PVL binding to polymorphonuclear leukocytes and impede the development of new pores [115]. A reduction in inflammatory reactions and tissue damage was also found in this non-infectious rabbit model of endophthalmitis, with the tetravalent anti-PVL antibody [115].

In various in vivo models, antibodies targeting superantigens were shown to neutralize these toxins and have been linked to protection [116]. The staphylococcal enterotoxin B (SEB) is one of the most studied enterotoxins, and its designation as a bioweapon makes it an interesting target to produce anti-toxin-neutralizing antibodies. Drozdowski et al. generated and selected human monoclonal antibodies (HuMAbs) specific for SEB from human B-cell hybridomas [117]. In vitro, these antibodies exhibited biological activity against SEB, and, HuMAb-154, which had the highest anti-SEB affinity, demonstrated both preventive and therapeutic action in a mouse model of SEB-induced mortality [117]. In addition, in numerous mouse models, including sepsis and cutaneous and deep tissue abscesses, another monoclonal antibody against SEB, named 20B1, was demonstrated to be protective against MRSA infection [118].

Therefore, all these therapy examples highlight the usefulness of antibodies as anti-virulence treatments able to neutralize staphylococcal toxins.

### 3.2. Nanoparticles

Aside from the use of anti-toxin antibodies in anti-virulence therapies, researchers have also shown the effectiveness of manufactured nanoparticles that imitate cell membranes, such as liposomes, in sequestering bacterial toxins in vitro and in vivo [119]. For example, Henry et al. demonstrated the ability of artificial liposomes to trap bacterial toxins in vitro while maintaining the integrity of mammalian cells [120]. They also discovered that administering artificial liposomes to mice during in vivo studies helped them recover from septicemia induced by *S. aureus,* as well as protect them against pneumonia [120]. Because customized liposomes are made entirely of naturally occurring lipids, they are not bactericidal and could be employed alone or in combination with antibiotics to treat bacterial infections and reduce toxin-induced tissue damage [120]. Interestingly, Wolfmeier et al. employed sphingomyelin liposomes, with or without cholesterol, to neutralize secreted PSMs and other virulence factors in vitro during human blood or epithelial cell staphylococcal infections, as well as in a murine dermonecrosis model [121]. Sphingomyelin liposomes blocked cell lysis by PSMs, particularly PSM3, whereas cholesterol-containing sphingomyelin liposomes preferentially trapped α-hemolysin [121]. A combination of both liposome types was recently evaluated in a Phase I clinical trial against severe pneumococcal pneumonia, although its utility in *S. aureus* pneumonia remains unknown. Furthermore, targeting both PSMs and α-hemolysin at the same time remains a possibility, as PSMs have been demonstrated to regulate α-hemolysin expression both in vitro and in vivo [23].

Recently, in response to *S. aureus* infection, exosomes (called “defensosomes”) with increased ADAM10 receptors were discovered to be produced from host cells in a TLR-dependent mode, resulting in α-hemolysin retention and a reduction in disease mortality [122]. Then, therapeutic poly (lactic-co-glycolic acid) (PLGA)-based nanoparticles covered with natural human erythrocyte membranes performed a comparable decoy effect to fight infection and reduce the activity of α-hemolysin [123].

Moreover, similar to other anti-virulence strategies, the nanoparticle-based neutralization and administration not only help to avoid severe bacterial infections but can also participate in reducing the development of antibiotic resistance [124].

### 3.3. RNAIII-Inhibiting Peptides

In addition to the direct neutralization strategy outlined in the previous sections, targeting *S. aureus* toxins can be carried out indirectly by affecting the regulatory processes that govern virulence genes’ expression. This approach is based on the utilization of small molecules, such as peptides, to target global regulators, such as the accessory gene regulator Agr in *S. aureus.* Agr regulates the quorum-sensing pathway that controls whether *S. aureus* develops a biofilm or remains planktonic, as well as toxin gene synthesis [44,125,126]. The P3 promoter of the *S. aureus* quorum-sensing Agr system transcribes RNAIII, a stable regulatory RNA that regulates the expression of a large variety of virulence factors [127]. Therefore, inhibiting RNAIII represents a promising strategy for reducing toxin expression as well as other virulence factors. When evaluated in cellulitis in in vivo models, RNAIII-inhibiting peptides (RIP) were found to block *agr* RNA transcripts and impede staphylococcal adhesion to mammalian cells, resulting in a decrease in *S. aureus* pathogenicity [128,129,130]. In an MRSA sepsis model, two new RIP derivatives were recently discovered to substantially extend mouse survival and reduce pathological damage without impacting bacterial viability [131]. Interestingly, in an *S. aureus*-induced sepsis mouse model, and in association with clinically prescribed antibiotics, RIP increased the healing of wounds and reduced mortality in comparison to antibiotics alone, thus confirming the potential of combined therapies [132,133]. Moreover, PSMs have been specifically targeted using a variety of methods to neutralize their pathogenic effect. In a mouse pneumonia model, targeting PSMs indirectly by inhibiting the Agr system with an RNAIII-inhibiting peptide resulted in a lower bacterial load and mortality [134].

### 3.4. Antimicrobial Peptides (AMPs)

Antimicrobial peptides (AMPs) have been known for several decades and are part of the innate immunity of practically all living organisms, ranging from bacteria, insects, and plants to vertebrates [135]. As of August 2021, the antimicrobial peptide database (https://aps.unmc.edu/ (accessed on 23 August 2021)) contains 3273 antimicrobial peptides from six kingdoms (369 bacteriocins/peptide antibiotics from bacteria, 5 from archaea, 8 from protists, 22 from fungi, 361 from plants, and 2424 from animals, including some synthetic peptide records). AMPs have a wide spectrum of antibacterial, antifungal, antiparasitic, and antiviral properties [135]. AMPs not only possess a large spectrum of antibacterial activity but can also display anti-toxin activities [136,137]. Cathelicidins and defensins are the two major categories of AMPs in humans. Human defensins are amphipathic cationic peptides that are divided into two types: *α*- and *β* -defensins, and, to date, four *α*-defensins have been identified from polymorphonuclear neutrophils (PMNs) [138]. Human neutrophil peptides (HNP1–HNP4) are part of the phagolysosome’s microbicidal machinery that can be detected in the extracellular environment after degranulation [139]. PVL is thought to have a role in CA-MRSA pathogenesis by attracting and lysing PMNs at the infection site, which induces tissue damage caused by the release of cytotoxic granule constituents [140]. Interestingly, Cardot-Martin et al. found that HNP3 defensins, but not HNP-1 or -2, substantially protect neutrophils from PVL-induced lysis by interacting with LukS-PV and LukF-PV, which disables PVL’s pore creation function and reduces PVL cytotoxic effects [141].

### 3.5. Natural Compounds

Natural product-based compounds that present anti-toxin properties correspond to a promising therapeutical approach to treat *S. aureus* infections [18]. A modified cyclodextrin compound, named IB201, is used to treat pneumonia. Cyclodextrins are cyclic oligosaccharides that are made from starch or starch derivatives. Because of its spatial resemblance to α-hemolysin, this compound was identified based on the prediction that it would prevent α-hemolysin action with a high affinity [142]. Moreover, in mice *S. aureus* pneumonia models, aloe-emodin, an active component from aloe vera, and apigenin, an active compound from parsley, both exhibited sufficient protection [143,144]. Other compounds, such as morin hydrate (also known as 2′,3,4′,5,7-pentahydroxyflavone), which is a flavonoid present in *Maclura pomifera* (Osage orange), in *Maclura tinctoria* (old fustic), and in the leaves of *Psidium guajava* (common guava), was discovered to disrupt the self-assembly of the transmembrane pore of α-hemolysin in a mouse model of pneumonia and then to decrease its hemolytic activity [145].

Oroxin A (ORA), oroxin B (ORB), and oroxylin A 7-O-glucuronide (OLG), three oroxylin glycosides, are natural flavonoids found in strawberries, grapes, onions, apples, Bignoniaceae plants, and other vegetables and fruits. These substances possess structural similarities and bind to hemolysin’s stem domain, preventing it from transitioning from monomer to oligomer in vitro and inhibiting its hemolytic action [146,147].

Friedman et al. showed that the pure olive chemical 4-hydroxytyrosol and the commercialized olive powder Hidrox-12, containing 6% of 4-hydroxytyrosol and 6% of additional phenolic compounds, were able to suppress the biological action of the superantigen enterotoxin A (SEA) [148]. However, this effect still remains to be validated in animal models.

Solonamide B, a cyclodepsipeptide from the halotolerant bacterium *Photobacterium halotolerans*, was one of the first natural inhibitors of the Agr signaling pathway to be reported [149]. Solonamide B and its derivatives prevent the quorum-sensing peptide AIP from interacting with AgrC. Interestingly, in CA-MRSA strains, such as USA300, solonamide B strongly reduced the activity of α-hemolysin and the transcription of *psma*-encoding PSMs resulting in an 80% reduction in toxicity of supernatants toward human neutrophils and rabbit erythrocytes [150,151,152].

Isorhamnetin, chrysin, and puerarin have been shown to inhibit RNAIII transcription and, hence, α-hemolysin expression, providing protection against MRSA and MSSA-induced pneumonia [153,154,155]. Isorhamnetin (also known as 3′-methoxy-3,4′,5,7-tetrahydroxyflavone) is an O-methylated flavanol found in apples, blackberries, cherries, and pears, as well as in medicinal plants and herbs [155]. Honey, propolis, the passion flowers *Passiflora caerulea* and *Passiflora incarnata*, and *Oroxylum indicum* all contain chrysin (5, 7-dihydroxyflavone) [156]. Puerarin is the main bioactive compound obtained from the root plant *Pueraria lobata Ohwi*, also called Gegen in traditional Chinese medicine [157].

Naringenin, a flavanone found primarily in grapefruit, but also in a range of fruits and herbs, has been shown to drastically lower the amounts of *agrA* and *hla* transcripts in *S. aureus* culture as well as to inhibit hemolysin synthesis and protect mice from *S. aureus*-induced pneumonia [158,159].

In vitro, *Castanea sativa* leaf extract 224C-F2 [160] and *Schinus terebinthifolia* berry extract 430D-F5 [161] were found to inhibit *agr* expression, thus resulting in decreased hemolysin synthesis and hemolytic activity. A pretreatment with 224C-F2 diminished infection-induced ulcer sizes and substantially lowered morbidity in an in vivo model of MRSA infection [160]. In addition, pretreatment with a single dose of 430D-F5 massively reduced skin ulcer formation and mortality in a mouse model of MRSA skin infection [161].

Ambuic acid is a fungal product that targets AgrB activity and has been shown to prevent hemolysin and RNAIII production in MRSA infections [162]. A single preventive administration of ambuic acid totally prevented skin ulcer formation in a murine mouse model [162]. Another fungal product, named omega-hydroxyemodin (OHM) and produced by *Penicillium restrictum*, decreases Agr activity by disrupting AgrA binding to its promoter. Importantly, OHM particularly inhibited the Agr pathway activation in a mouse model of MRSA cutaneous infection without affecting the host [163,164].

Even though some of the molecular mechanisms of these natural compounds have yet to be determined, they provide possible novel scaffolds for the development of successful anti-virulence therapeutics toward *S. aureus* infection.

### 3.6. Vaccines

Despite numerous attempts, there is currently no vaccination against *S. aureus*. As mentioned in the first section, *S. aureus* secretes a broad range of toxins during colonization and infection of the host, which poses a challenge for vaccine development.

There have been several studies to investigate the efficacy of α-hemolysin as a vaccine agent. Indeed, H35L, a mutant isoform of α-hemolysin, was discovered to have minimal hemolytic action [165]. In a mouse pneumonia model, this inactivated toxin (toxoid) was studied in two separate models and found to be efficacious through both active vaccination and the development of protective rabbit anti α-hemolysin antibodies; despite this fact, no human trials have been conducted to date [166].

IBT-VO2 is a multivalent vaccine currently under investigation. α-hemolysin, PVL LukS, LukF, LukAB, enterotoxins A and B, and toxic shock syndrome toxin 1 toxoids are all included in this heptavalent vaccine [167]. The multi-subunit vaccine generates an antibody response that is cross-reactive with 12 to 15 *S. aureus* toxins and gives protection in different mice and rabbit infection models due to structural similarities [167]. Recently, IBT-VO2 entered a Phase I clinical study after completing the encouraging pre-clinical phase, and as a result, received additional funding to help it progress.

Previous studies in animal models suggested that PVL subunits could be useful vaccines, but these attempts have yet to be converted into human trials [168,169]. However, for the creation of the StaphVax vaccine, recombinant PVL subunits were exploited (Nabi Biopharmaceuticals, Alpharetta, GA), but this vaccine failed in Phase 3 clinical testing [170]. However, some of the vaccine antigens, including PVL, were recycled into a new vaccine called PentaStaph, which was acquired by GlaxoSmithKline Biologicals (GSK) [171,172].

In mice, an inactive isoform of the staphylococcal enterotoxin B (SEB) was cloned into an *Lactococcus lactis* strain and tested as an oral vaccine. The vaccination was able to generate a significant antibody response, thus resulting in improved survival of infected mice [173]. Another vaccine against this SEB toxin, named STEBVax, was also generated and corresponds to a recombinant isoform that impedes the toxin from interacting with the major histocompatibility complex (MHC) class II [174]. If effective, this vaccine could be useful as a polyvalent *S. aureus* vaccine in general.

In another study, the injection of TSST-1-specific antibodies to treat toxic shock syndrome has been found to reduce mortality in a septic mouse model of infection [175]. Moreover, a modified TSST-1 antigen was also exploited to create an attenuated TSST-1 vaccine able to prevent infection during sepsis in mice [175]. Subsequently, a recombinant TSST-1 variant vaccine was created and tested in a Phase I clinical study, which was quite well tolerated, and a Phase II trial was engaged [176,177].

Even though numerous vaccine candidates have demonstrated protective efficacy in preclinical or early clinical investigations as detailed above, no vaccine has been authorized to date for human use.

### 3.7. Others

Eukaryotes, archaea, and bacteria create extracellular vesicles (EVs), which are lipid bilayers that form lumen-containing spheres with diameters ranging from 20 to 500 nm. EVs contain a variety of proteins, polysaccharides, nucleic acids, and lipids. EVs from Gram-positive bacteria carry physiologically active toxins, display cytotoxicity, and stimulate proinflammatory mediators, thus having a significant role in host–pathogen interactions [178]. Unfortunately, the toxicity of staphylococcal EVs limited their use as a vaccine platform. However, in a recent study, Wang et al. engineered EVs with unique features in the *S. aureus* USA300 strain, representative of the dominant CA-MRSA clone in the United States [179]. The originality of this study was to consider that *S. aureus* EVs could be used as a vaccine platform if their cytotoxicity was reduced. Therefore, EVs over producing Hla- and LukE-modified toxins that possess the capacity to be immunogenic without being toxic were engineered in order to stimulate the production of toxin-neutralizing antibodies. Immunization with engineered EVs showed considerable protection in an *S. aureus* lethal sepsis model [179]. Though the efficiency of these vesicles as a novel vaccine platform against different *S. aureus* strains and in additional infection models has yet to be determined, they do represent an attractive promise.

Moreover, novel approaches have been developed to combat enterotoxins. In an in vitro T-cell experiment, Mattis et al. developed a yeast display technology to create a soluble T-cell receptor variable domain variation capable of neutralizing both SEC and SEB enterotoxins. In different rabbit models, including endocarditis and necrotizing pneumonia, this variation was proven to be effective in reducing the infection [180].

**Table 2:** Summary of the anti-toxin treatments strategies.

Treatment	Name	Target	References
Antibodies	MEDI4893 (suvratoxumab)	α-hemolysin	[98,99,100]
	AR-301	α-hemolysin	[106]
	ASN100	α-hemolysin, Panton–Valentine leukocidin (PVL), LukAB, Ɣ-hemolysin AB (HlgAB), Ɣ-hemolysin CB (HlgCB), leukocidin ED (LukED)	[107]
	LTM14	α-hemolysin	[99]
	IVIg	α-hemolysin, PVL	[112,113,114]
	HuMAb-154	Staphylococcal enterotoxin B (SEB)	[117]
	20B1	Staphylococcal enterotoxin B (SEB)	[118]
Nanoparticles	Sphingomyelin liposomes	Phenol-soluble modulins (PSMs)	[121]
	Cholesterol-containing sphingomyelin liposomes	α-hemolysin	[121]
	Poly (lactic-co-glycolic acid) (PLGA)-based nanoparticles covered with natural human erythrocyte membranes	α-hemolysin	[123]
RNAIII-inhibiting peptides	RNAIII-inhibiting peptides (RIP)	*agr* RNA transcripts	[128,129,130]
Antimicrobial peptides	HNP3	PVL	[141]
Natural compounds	Cyclodextrin IB201	α-hemolysin	[142]
	Aloe-emodin	α-hemolysin	[144]
	Apigenin	α-hemolysin	[143]
	Morin hydrate (2′,3,4′,5,7-pentahydroxyflavone)	α-hemolysin	[145]
	Oroxylin glycosides (oroxin A (ORA), oroxin B (ORB), and oroxylin A 7-O-glucuronide (OLG))	α-hemolysin	[146,147]
	4-hydroxytyrosol Hidrox-12	Staphylococcal enterotoxin A (SEA)	[148]
	Solonamide B	Quorum-sensing peptide (AIP)	[149,150,151,152]
	Isorhamnetin (3′-methoxy-3,4′,5,7-tetrahydroxyflavone)	α-hemolysin	[155]
	Chrysin (5, 7-dihydroxyflavone)	α-hemolysin	[156]
	Puerarin	α-hemolysin	[157]
	Naringenin	*agrA* and *hla* expression	[158,159]
	224C-F2 (*Castanea sativa* leaf) 430D-F5 (*Schinus terebinthifolia* berry)	*agr* expression	[161]
	Ambuic acid	AgrB activity, *RNAIII* expression	[162]
	Omega-hydroxyemodin (OHM)	AgrA	[163,164]
Vaccines	H35L	α-hemolysin	[165,166]
	IBT-VO2	α-hemolysin, PVL, enterotoxins A and B, toxic shock syndrome toxin 1 (TSST-1)	[167]
	StaphVax	PVL	[170]
	STEBVax	SEB	[174]
	Attenuated TSST-1 vaccine	TSST-1	[175,176,177]
Others	Extracellular vesicles (EVs)	α-hemolysin, LukE	[181]
	Yeast display technology to create a soluble T-cell receptor	SEC, SEB	[180]

## 4. Conclusions and Future Directions

The problem of antibiotic resistance has prompted scientists around the world to explore alternatives for effective treatments. Because antimicrobial resistance is a complex phenomenon, the solution to this problem comprises a variety of techniques aimed at reducing the factors that contribute to the establishment of resistance and spread. These strategies require the development of novel therapeutic drugs that work on principles distinct from those currently available for antibiotics. Bacterial toxins, as detailed in this review, are directly involved in disease outcomes. Anti-toxin therapies have been proposed as a promising alternative in this regard, with the intention of reducing pathogen virulence without exposing pathogens to selective pressure.

Anti-toxin therapies target diseases that are the most dangerous to patients, such as hospital-acquired bacterial pneumonia and ventilator-associated bacterial pneumonia, osteomyelitis, sepsis, and endocarditis, and have the capacity to enhance chances of survival. One of the benefits of these anti-toxin treatments is their use in conjunction with antibiotics to help fight the most dangerous infections. Furthermore, anti-toxin therapies contribute to significantly reduce the bacterial load, most likely by interfering with the bacterial strategies used to multiply involving secreted toxins [182]. Moreover, anti-toxin treatments do not place selective pressure on bacterial growth since they neutralize the pathogen rather than killing it, which could provide a long-term solution to the resistance issue. However, the potential of anti-toxin treatments to counter drug resistance without putting severe selective pressure on the bacterial population needs more investigation.

Even though efforts to develop innovative anti-toxin compounds have had varying levels of success, the potential leads need pharmacology and toxicology evidence. For extensive mechanistic study, additional studies should concentrate on a few very promising candidates. The anti-toxin treatments potential negative effects must also be considered. Because of the toxins’ extensive combination and cross-reactivity, efforts to interfere at the host–toxin level necessitate robust anti-toxin efficacy. Therefore, staphylococcal toxin biology requires more research to decipher the specific toxin roles, the differences in expression or genetic existence of toxins all over strain lineages, and the importance of specific toxins along various clinical strains. Another drawback of anti-toxin compounds is their possible limited spectrum efficacy, as these treatment candidates only specifically target virulence-mediated pathways in certain *S. aureus* strains, thereby limiting their general clinical use.

Moreover, it is clear that evaluating the effectiveness of anti-toxin compounds is delayed by the lack of therapy models, which may more precisely mimic the clinical condition in humans. Thus, developing such models represents an essential future direction. Human clinical trials will always be required to prove the success of a treatment. However, even though animal models remain necessary to decipher fundamental host–pathogen interactions and even though many potentially promising *S. aureus* anti-toxin therapeutics exist, most have failed in human trials or have not been tested. Therefore, the development of humanized mice with engrafted human immune cells for instance could help improve the translatability of animal investigations to human trials in the future [183,184,185]. This strategy will improve animal models, thus helping in deciding which treatments should proceed to clinical trials.

It is also necessary to specify which criteria will be used to assess the anti-virulence therapy’s efficacy, as well as which types of infections the treatment is most suited for. For instance, a defective Agr–quorum sensing system appears to be favorable for the pathogen in *S. aureus* chronic infections or bacteremia [186]. Furthermore, it was recently demonstrated that a dysfunctional Agr system could facilitate antibiotic resistance to gentamicin and ciprofloxacin [187]. Moreover, phenol-soluble modulin toxins are known to be implicated in the regulation of *S. aureus* persister cell populations [188]. Then, as the Agr system oversees PSMs’ production, a misfunctioning system is likely to suppress PSM expression, favoring the formation of persister cells resistant to antibiotics.

To summarize, a tremendous amount of work has investigated *S. aureus* toxins, expanding our understanding of their mode of action and involvement in pathogenesis, and several promising therapies have resulted from various treatment strategies. However, improved therapeutical models need to be developed to validate most of these anti-toxin treatments.
